# Selection of Reference Genes for RT-qPCR Analysis in *Coccinella septempunctata* to Assess Un-intended Effects of RNAi Transgenic Plants

**DOI:** 10.3389/fpls.2016.01672

**Published:** 2016-11-08

**Authors:** Chunxiao Yang, Evan L. Preisser, Hongjun Zhang, Yong Liu, Liangying Dai, Huipeng Pan, Xuguo Zhou

**Affiliations:** ^1^College of Plant Protection, Hunan Agricultural UniversityHunan, China; ^2^Institute of Plant Protection, Hunan Academy of Agricultural SciencesHunan, China; ^3^Department of Entomology, University of Kentucky, LexingtonKY, USA; ^4^Department of Biological Sciences, University of Rhode Island, KingstonRI, USA; ^5^Institute for the Control of Agrochemicals, Ministry of AgricultureBeijing, China; ^6^Department of Entomology, South China Agricultural University, Key Laboratory of Bio-Pesticide Innovation and Application of Guangdong ProvinceGuangzhou, China

**Keywords:** *Coccinella septempunctata*, RT-qPCR, reference gene, RNAi transgenic plants, environmental risk assessment, plant incorporated protectant

## Abstract

The development of genetically engineered plants that employ RNA interference (RNAi) to suppress invertebrate pests opens up new avenues for insect control. While this biotechnology shows tremendous promise, the potential for both non-target and off-target impacts, which likely manifest via altered mRNA expression in the exposed organisms, remains a major concern. One powerful tool for the analysis of these un-intended effects is reverse transcriptase-quantitative polymerase chain reaction, a technique for quantifying gene expression using a suite of reference genes for normalization. The seven-spotted ladybeetle *Coccinella septempunctata*, a commonly used predator in both classical and augmentative biological controls, is a model surrogate species used in the environmental risk assessment (ERA) of plant incorporated protectants (PIPs). Here, we assessed the suitability of eight reference gene candidates for the normalization and analysis of *C. septempunctata v-ATPase A* gene expression under both biotic and abiotic conditions. Five computational tools with distinct algorisms, *geNorm, Normfinder, BestKeeper*, the Δ*C*_t_ method, and *RefFinder*, were used to evaluate the stability of these candidates. As a result, unique sets of reference genes were recommended, respectively, for experiments involving different developmental stages, tissues, and ingested dsRNAs. By providing a foundation for standardized RT-qPCR analysis in *C. septempunctata*, our work improves the accuracy and replicability of the ERA of PIPs involving RNAi transgenic plants.

## Introduction

RNA interference (RNAi)-based genetically modified (GM) plants targeting insects have been developed and offer a new approach for insect control (Baum et al., 2007; Mao et al., 2007; Zha et al., 2011). Consuming transgenic maize that expresses an internal housekeeping gene, *vacuolar ATPase subunit A*, for example, significantly increases *Diabrotica virgifera virgifera* larval mortality (Baum et al., 2007). Technical and regulatory hurdles notwithstanding (Lundgren and Duan, 2013), commercialization of transgenic maize that expresses long dsRNAs for *D. v. virgifera* control appears likely in the near future (Kupferschmidt, 2013; Palli, 2014; Petrick et al., 2016; Tan et al., 2016). The impact of RNAi transgenic crops on non-target organisms (NTOs) is a major environmental concern. These NTOs include biological control agents that play essential role in integrated pest management (Romeis et al., 2008; Roberts et al., 2015; Xu et al., 2015).

*Coccinella septempunctata* (Coleoptera: Coccinellidae), the seven-spotted ladybeetle, is a generalist predator used for classical and augmentative biological control in many cropping systems. Its larvae and adults are voracious arthropod predators that also feed on pollen, nectar, and petals (Kalushkov and Hodek, 2004). *C. septempunctata* has been widely used as a ‘model’ NTO to evaluate the potential risks of *Bacillus thuringiensis* (Bt) transgenic crops (Harwood et al., 2005, 2007; Alvarez-Alfageme et al., 2012). The mode of action of RNAi transgenic plants suggests that un-intended effects will likely occur via altered gene expression in NTOs (Berezikov, 2011), and reverse transcriptase-quantitative polymerase chain reaction (RT-qPCR) provides an important tool for detecting such changes. The importance of systematic criteria for selecting and evaluating reference genes used in RT-qPCR studies (Kalushkov and Hodek, 2004; Bustin et al., 2013; Li et al., 2013; Sinha and Smith, 2014; Zhu et al., 2014; Pan et al., 2015a; Wang et al., 2015) is illustrated by the fact that using one or multiple unsuitable reference genes for normalization can produce up to 20-fold differences in expression values and misinterpret gene expression results (Bustin et al., 2013; Hellemans and Vandesompele, 2014). Since RNAi-based insecticides and/or RNAi transgenic plants may cause lethal or sublethal effects in NTOs through changes in gene expression, RT-qPCR analysis without rigorous reference gene selection and validation may lead to inaccurate assessment of risks.

Reverse transcriptase-quantitative polymerase chain reaction is a powerful method for quantifying gene expression (Vandesompele et al., 2002). Although RT-qPCR is extensively used for measuring transcript abundance, variation in RNA extraction, RNA integrity and quality, enzymatic efficiency, and PCR efficiency can influence *C*_q_-values (Bustin et al., 2005; Strube et al., 2008). RT-qPCR generally involves normalization to the expression of a suite of appropriated reference genes in parallel. Even though reference gene transcript levels should ideally be stable across a range of different conditions, many commonly used reference genes differ dramatically across treatments (Kalushkov and Hodek, 2004; Bustin et al., 2013; Li et al., 2013; Sinha and Smith, 2014; Zhu et al., 2014; Pan et al., 2015a; Wang et al., 2015). Such high level of variation emphasizes the need to determine stable reference genes for RT-qPCR analyses on a case-by-case basis, even for the same species.

The goal of the current study is to select suitable reference genes for RT-qPCR analysis in *C. septempunctata*, specifically, for the normalization and analysis of *vacuolar-type H*^+^*-ATPase* (*V-ATPase*), a potential molecular target for RNAi transgenic plants. A total of eight candidate reference genes were investigated: *NADH dehydrogenase subunit 5* (*NADH*), *elongation factor 1*α (*EF1A*), β*-actin* (*Actin*), α*-tubulin* (*Tubulin*), *arginine kinase* (*ArgK*), *28S ribosomal RNA* (*28S*), *16S ribosomal RNA* (*16S*), and *18S ribosomal RNA* (*18S*). The consistency of each reference gene was evaluated under one abiotic (dietary RNAi) and two biotic (developmental stage and tissue) conditions. Expression of the target gene, *V-ATPase*, was investigated under each of the three treatments using different normalization strategies.

## Materials and Methods

### Insects

*Coccinella septempunctata* (Coleoptera: Coccinellidae) pupae were collected from alfalfa at the north farm of University of Kentucky in June, 2015. Larvae and adults were reared in the laboratory at 23 ± 0.5°C temperature, 16L: 8D photoperiod, and 50% relative humidity. They were provisioned with pea aphids (*Acyrthosiphon pisum*) reared in a greenhouse at 20–28°C on fava bean, *Vicia faba* (Fabales, Fabaceae).

#### Biotic Factors

All developmental stages of *C. septempunctata* were sampled: eggs, all four larval instars (collected at the first day of each instar), pupae, female and male adults. Different body tissues, including the head, gut, and carcass (body without head or viscera), were dissected from larvae.

#### Abiotic Factor

For the dietary RNAi treatment, first-instar *C. septempunctata* larvae were supplied with 15% sugar solution containing one of the following: (1) *in vitro* synthesized dsRNAs from a target gene, *C. septempunctata V-ATPase subunit A* (dsCS) (dsCS forward: TAATACGACTCACTATAGGGAGATCTCTTTTCCCATGT; dsCS reverse: TAATACGACTCACTATAGGGAGAGCATCTCGGCCAGAC); (2) a specific positive control, *V-ATPase A* 400 bp fragment amplified from *D. v. virgifera*; (3) a control gene, β*-glucuronidase* (dsGUS); and (4) a blank control, H_2_O (Yang et al., 2015). Neonate larvae emerging from their eggs were kept in separate petri dishes for 2 days; each larvae was provisioned on a daily basis with a 2 μl droplet containing 4 μg/μl dsRNA or the water control. On days 3, 5 individuals per treatment were collected for RT-qPCR analysis.

The number of sampled individuals per replicate in the developmental stage study was as follows: Egg stage: 15 eggs; first instar: five individuals; second instar: five individuals; third instar: three individuals; fourth instar: one individual; pupal: one pupa; adult male or female stage: one male or female individual. For the other biotic and abiotic factors, five individuals were sampled per replicate, and each experiment was replicated three times. Samples were placed in 1.5 ml centrifuge tubes, quickly frozen in liquid nitrogen, and stored at -80°C prior to total RNA isolation.

### Total RNA Extraction and cDNA Synthesis

Total RNA was isolated using TRIzol reagent (Invitrogen, Carlsbad, CA, USA) in accordance with previously published methods (Yang et al., 2014, 2015). DNase treated total RNA was denatured at 75°C for 5 min and immediately chilled on ice. The concentration of RNA was determined using a NanoDrop 2000c Spectrophotometer. RNA concentrations were as follows: 287.9 ± 87.0 ng/μl [mean ± standard error of the mean (SEM)] for eggs, 392.0 ± 45.1 ng/μl for the first instars, 854.0 ± 62.2 ng/μl for the second instars, 480.4 ± 12.9 ng/μl for the third instars, 879.2 ± 153.8 ng/μl for the fourth instars, 727.8 ± 147.1 ng/μl for pupae, 622.6 ± 109.1 ng/μl for male adults, 558.1 ± 78.7 ng/μl for female adults, 501.8 ± 72.5 ng/μl for heads, 1095.5 ± 39.4 ng/μl for carcasses, 597.6 ± 62.5 ng/μl for guts, and 457.9 ± 29.7 ng/μl for dsRNA experimental samples. The OD260/280 ratio of all samples was 1.9–2.1. Single-stranded cDNA was synthesized for each biological sample from 1.0 μg of total RNA using the M-MLV reverse transcription kit (Invitrogen, Carlsbad, CA, USA) and a random N primer (NNNNNN). The cDNA was diluted 10X for the subsequent RT-qPCR studies.

### Double-Stranded RNA Preparation

First-strand cDNA was prepared using 2.0 μg of total RNA with the M-MLV reverse transcription kit (Invitrogen, Carlsbad, CA, USA) following the manufacturer’s recommendations. Pair-wise comparison showed that the entire coding sequence of *v-ATPase A* from *D. v. virgifera* and *C. septempunctata* share a 81.0% nucleotide sequence similarity (**Supplementary Figure S1**). The 400 bp region with the highest sequence similarity (85%) was selected as the template to synthesize arthropod-active dsRNAs (**Supplementary Figure S2**). A non-specific negative control, the β*-glucuronidase* (GUS) gene was cloned into pBTA2 vector and PCR amplified using gene specific primers, which amplified a 560 bp fragment containing T7 polymerase promoter region at the 5′ end. PCR amplifications were performed in 50 μl reactions containing 10 μl 5 × PCR Buffer (Mg2+ Plus), 1.0 μl dNTP mix (10 mM of each nucleotide), 5.0 μl of each primer (10 μM each), and 0.25 μl of GoTaq (5 u/μl) (Promega). The PCR parameters were as follows: one cycle of 94°C for 3 min; 35 cycles of 94°C for 30 s, 59°C for 45 s and 72°C for 1 min; a final cycle of 72°C for 10 min. The PCR product was used as template to generate dsRNA with the T7 MEGAscript kit (Ambion, Austin, TX, USA) following the manufacturer’s protocol. The synthesized dsRNAs were suspended in nuclease-free H_2_O and quantified with a NanoDrop 2000c spectrophotometer and then stored at -20°C.

### Gene Cloning and Primer Design

This study assessed eight reference genes previously commonly used in RT-qPCR analyses and that have been verified as stable reference genes in other species (**Table 1**). Primers for *18S* (AY748147), *28S* (DQ202668), *16S* (JX896437), and *NADH* (JQ321839) were designed according to the sequences downloaded from NCBI. Degenerate primers for the remaining four reference genes including *Actin, ArgK, EF1A, Tubulin*, and one target gene *V-ATPase* were designed with CODEHOP^1^ based on conserved amino acid residues among the Coleopteran insect species (Supplementary Table S1). PCR amplifications were performed in 50 μl reactions containing 10 μl 5 × PCR Buffer (Mg2+ Plus), 1 μl dNTP mix (10 mM of each nucleotide), 5 μl of each primer (10 μM each), and 0.25 μl of GoTaq (5 u/μl) (Promega). The PCR parameters were as follows: one cycle of 94°C for 3 min; 35 cycles of 94°C for 30 s, 59°C for 45 s and 72°C for 1 min; a final cycle of 72°C for 10 min. Amplicons of the expected sizes were purified, cloned into the pCR4-TOPO vector (Invitrogen, Carlsbad, CA, USA), and sent out for sequencing. The confirmation of the reference gene was done by sequence analysis (Supplementary Data Sheet 1), the primers (**Table 1**) used for RT-qPCR were designed online^2^ using previously described parameters (Yang et al., 2015).

**Table 1:** Primers used for reverse transcriptase-quantitative polymerase chain reaction (RT-qPCR).

Gene	Primer sequences (5′–3′)	Length (bp)	Efficiency (%)	*R*^2^	Linear regression
*18S*	F:CCAGTAAGCGCGAGTCATAA	103	99.6	0.9975	*y* = -3.3323x + 13.778
	R: GGTCCGAAGACCTCACTAAATC				
*28S*	F:TCGAAACGACCTCAACCTATTC	93	113.0	0.9869	*y* = -3.0462x + 12.955
	R: TTGGCACTCTGACCGAAATC				
*16S*	F: GGACCTGCCCACTGAATTATTA	106	97.6	0.9992	*y* = -3.3805x + 15.749
	R: TTCTCATCAAACCATTCATACAAGC				
*EF1A*	F: CCTGAAGTGGAAGACGAAGAG	98	106.1	0.9782	*y* = -3.1839x + 20.008
	R: AGAAGGAAAGGCTGATGGTAAA				
*NADH*	F: AGTAAGAGGAGTAAAGGCATGAAA	75	91.2	0.9961	*y* = -3.5523x + 20.036
	R:CCTATATGGTTGATTGATGATAAGGC				
*Actin*	F: GCGTAACCTTCGTAGATTGGTA	100	102.5	0.9994	*y* = -3.2646x + 19.117
	R: CAAGCTGTACTCTCCCTGTATG				
*Tubulin*	F: ACAGGTTTCAAAGTGGGTATCA	102	101.6	0.9994	*y* = -3.2836x + 23.82
	R: GGTGGTGTTTGACAACATGC				
*ArgK*	F: GTCGAGCTTAGCCTTGTTAGAG	103	110.6	0.9661	*y* = -3.091x + 23.19
	R: GCTGGGTTTCCTCACTTTCT				
*V-ATPase*	F: CCTCAAGGTACACCTCCAATTC	84	91.9	0.9935	*y* = -3.5346x + 20.23
	R: AGCTAATGTTCCAGGACGAATAA				

### Reverse Transcriptase-Quantitative Polymerase Chain Reaction (RT-qPCR)

Details regarding RT-qPCR amplifications and programs were provided in Yang et al. (2014); briefly, PCR reactions (20 μl) contained 7.0 μl of ddH_2_O, 10.0 μl of 2× SYBR Green Master Mix (Bio-Rad), 1.0 μl of each specific primer (10 μM), and 1.0 μl of first-strand cDNA template. Reactions occurred in 96-well format Microseal PCR plates (Bio-Rad) in triplicate. Reactions were performed in a MyiQ single Color Real-Time PCR Detection System (Bio-Rad). The standard curve for each candidate was generated from cDNA serial dilutions (1/5, 1/25, 1/125, 1/625, and 1/3125). The corresponding RT-qPCR efficiencies (E) were expressed in percentage according to the equation: *E* = (10^[-1/slope]^-1) × 100. The coefficients of determination (*R*^2^) for a linear regression model with one independent variable was obtained according to the method in excel.

### Determination of Reference Gene Expression Stability

The reference gene expression was evaluated using *geNorm* (Vandesompele et al., 2002), *NormFinder* (Andersen et al., 2004), *BestKeeper* (Pfaﬄ et al., 2004), and the Δ*C*_t_ method (Silver et al., 2006). Candidate reference genes were analyzed with *RefFinder*^3^ (Xie et al., 2012). Optimal reference gene number for target gene normalization was determined by pairwise variation (*V*_n_/*V*_n+1_) (0.15 recommended threshold); *V*-values were computed by *geNorm* (Vandesompele et al., 2002).

### Reference Gene Validation

For the tissue and dietary RNAi experiments, reference gene reliability was assessed by normalizing *V-ATPase* expression profiles with the two most- and two least-stable non-rRNA genes. For the development experiment, reference gene reliability was assessed by normalizing *V-ATPase* expression profiles with the three most- and three least-stable non-rRNA genes. Relative gene expression of *V-ATPase* was calculated using the 2^-ΔΔ^*^Ct^* method (Livak and Schmittgen, 2001). One-way ANOVA was used to compare *V-ATPase* expression under each dietary RNAi treatments, across different developmental stages, and in different tissue types.

## Results

### Candidate Gene Cloning and Performance

Four reference genes (*Actin, ArgK, EF1A, and Tubulin*) and one target gene (*V-ATPase*) were cloned based on degenerate primers. All candidate genes were expressed in *C. septempunctata* and visualized by a single amplicon of the expected size (**Supplementary Figure S3**). Gene-specific amplification of all candidate genes was confirmed by a single peak in melt-curve analysis (**Supplementary Figure S4**). The PCR efficiency (E), correlation coefficient (*R*^2^), and linear regression equation characterizing each standard curve were given in **Table 1**. The standard curve of each gene was also provided (**Supplementary Figure S5**).

**FIGURE 1:**
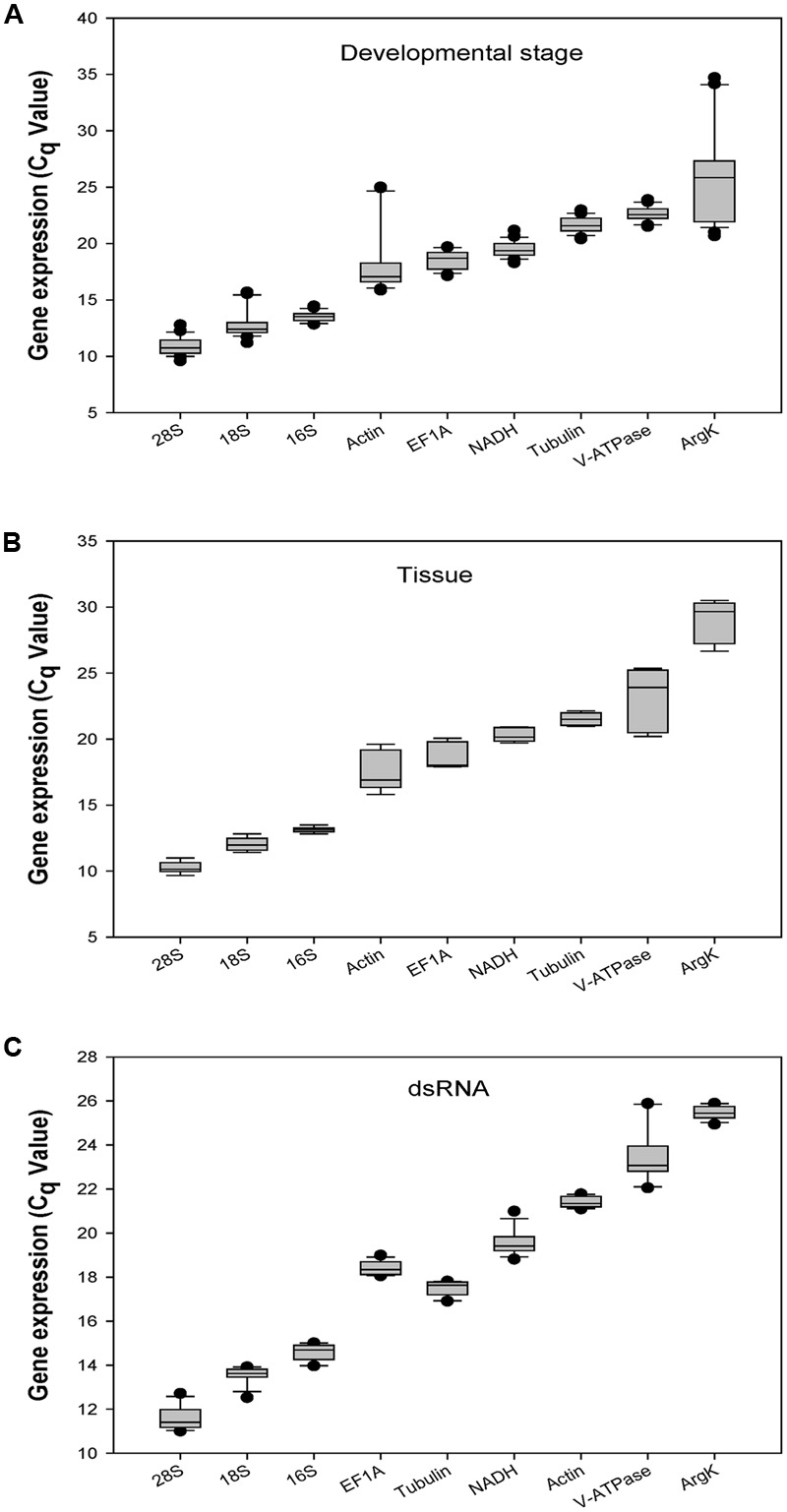
**Expression profiles of the eight candidate reference genes and one target gene in all three experiments in *Coccinella septempunctata*.** The whiskers indicate the standard error of the mean. **(A–C)** indicated the gene expression values under developmental stage, tissue, and dsRNA experimental conditions, respectively

The *C*_q_-values of all candidate genes under the three experimental conditions ranged from 10 to 29. The three ribosomal genes (*18S, 28S*, and *16S*) showed mean *C*_q_-values less than 15 cycles. *Actin, EF1A, Tubulin, NADH*, and *V-ATPase* had *C*_q_-values ranging from 17 to 24 cycles. *28S* and *ArgK* were the most- and least-expressed reference genes, respectively (**Figure 1**).

### Expression Stability of the Reference Genes under Different Experimental Conditions

*geNorm* calculates the expression stability value ‘M’ for each reference gene. In the developmental stage study, *16S* was the most stable gene. In different tissues, *18S* and *28S* were the most stable genes. In the dsRNA treatment, *18S* and *Tubulin* were the most stable genes. **Table 2** provides *geNorm-*based reference gene stability sequences for each experimental condition.

**Table 2:** Stability of candidate reference gene expression under different experimental conditions.

Conditions	CRGs^∗^	*geNorm*		*Normfider*		*BestKeeper*		Δ*C*_t_	
		Stability	Rank	Stability	Rank	Stability	Rank	Stability	Rank
Developmental	*18S*	1.032	6	0.154	1	0.876	6	1.658	4
Stage	*28S*	0.901	5	0.409	2	0.620	4	1.560	2
	*16S*	0.668	1	0.710	3	0.338	1	1.459	1
	*EF1A*	0.720	2	1.429	6	0.734	5	1.765	6
	*NADH*	0.819	4	0.806	4	0.581	3	1.592	3
	*Actin*	1.440	7	2.209	7	1.893	7	2.697	7
	*Tubulin*	0.743	3	1.145	5	0.578	2	1.670	5
	*ArgK*	1.997	8	3.815	8	3.041	8	3.947	8
Tissue	*18S*	0.350	1	0.175	1	0.372	3	1.043	4
	*28S*	0.350	1	0.175	1	0.350	2	1.040	3
	*16S*	0.423	2	0.210	2	0.157	1	0.996	1
	*EF1A*	0.597	5	0.830	3	0.845	6	1.287	6
	*NADH*	0.464	3	0.210	2	0.398	5	1.020	2
	*Actin*	0.922	6	1.731	4	1.249	7	1.908	7
	*Tubulin*	0.498	4	0.210	2	0.389	4	1.055	5
	*ArgK*	1.134	7	1.793	5	1.292	8	1.978	8
dsRNA	*18S*	0.335	1	0.354	5	0.232	2	0.593	5
	*28S*	0.524	7	0.609	8	0.406	8	0.761	8
	*16S*	0.442	5	0.392	6	0.322	6	0.627	6
	*EF1A*	0.393	3	0.200	1	0.258	4	0.563	2
	*NADH*	0.478	6	0.462	7	0.382	7	0.684	7
	*Actin*	0.374	2	0.289	3	0.200	1	0.558	1
	*Tubulin*	0.335	1	0.287	2	0.259	5	0.576	3
	*ArgK*	0.412	4	0.294	4	0.241	3	0.584	4

*^∗^Candidate reference genes.*

*NormFinder* calculates the expression stability value ‘SV’ for each reference gene. In the developmental stage study, *18S* was the most stable gene. In different tissues, *28S* and *18S* were the most stable genes. In the dsRNA treatment, *EF1A* was the most stable gene. **Table 2** provides *NormFinder-*based reference gene stability sequences for each experimental condition.

*BestKeeper* calculates the expression stability value ‘SD’ for each reference gene. For the developmental stage and tissue experiments, *16S* was the most stable gene. For the dsRNA treatment, *Actin* was the most stable gene. **Table 2** provides *BestKeeper-*based reference gene stability sequences for each experimental condition.

The Δ*C*_t_ method identifies potential reference genes by comparing expression of reference gene pairs within each sample. For the developmental stage and tissue experiments, *16S* was the most stable gene. For the dsRNA treatment, *Actin* ranked as the most stable gene. **Table 2** provides Δ*C*_t_-based reference gene stability sequences for each experimental condition.

### Comprehensive Ranking of Expression Stability

**FIGURE 2:**
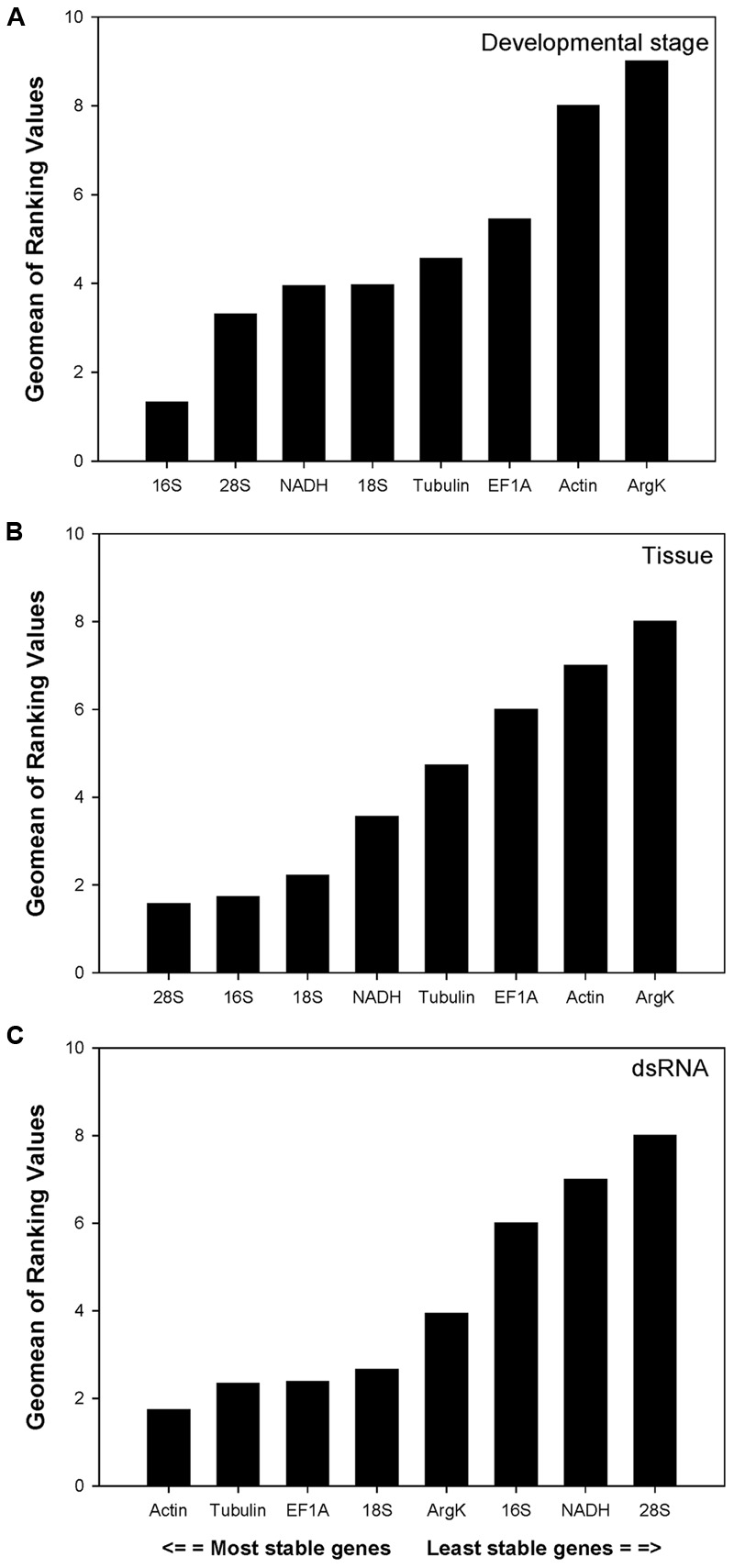
**Stability of candidate reference genes expression in *C. septempunctata* under different treatment according to their stability value by *RefFinder*.** A lower *Geomean* value indicates more stable expression. **(A–C)** indicated the gene stability values under developmental stage, tissue, and dsRNA experimental conditions, respectively.

For the development study, the integrated reference gene rankings (calculated using *RefFinder)* from most to least stable were as follows: *16S, 28S, NADH, 18S, Tubulin, EF1A, Actin*, and *ArgK* (**Figure 2A**). In different tissues, the integrated reference gene rankings were: *28S, 16S, 18S, NADH, Tubulin, EF1A, Actin*, and *ArgK* (**Figure 2B**). For the RNAi treatment, the integrated reference gene rankings were: *Actin, Tubulin, EF1A, 18S, ArgK, 16S, NADH*, and *28S* (**Figure 2C**).

### Optimal Number of Candidate Reference Genes According to *geNorm*

**FIGURE 3:**
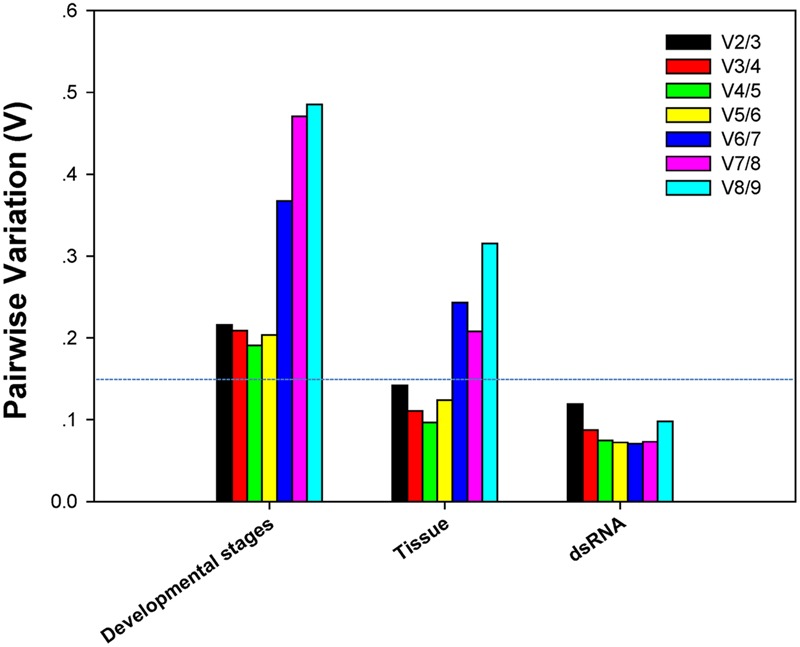
**Pairwise variation (V) values in three groups using *geNorm***.

*V*-values across the different developmental stages, although never lower than 0.15, were lowest at V3/4. This implies that three reference genes were sufficient for normalization throughout developmental stages (**Figure 3**). The first *V*-value less than 0.15 emerged at V2/3 in both the tissue and dsRNA experiments, suggesting that two reference genes were sufficient for normalization (**Figure 3**).

### Relative Gene Expression of *V-ATPase*

**FIGURE 4:**
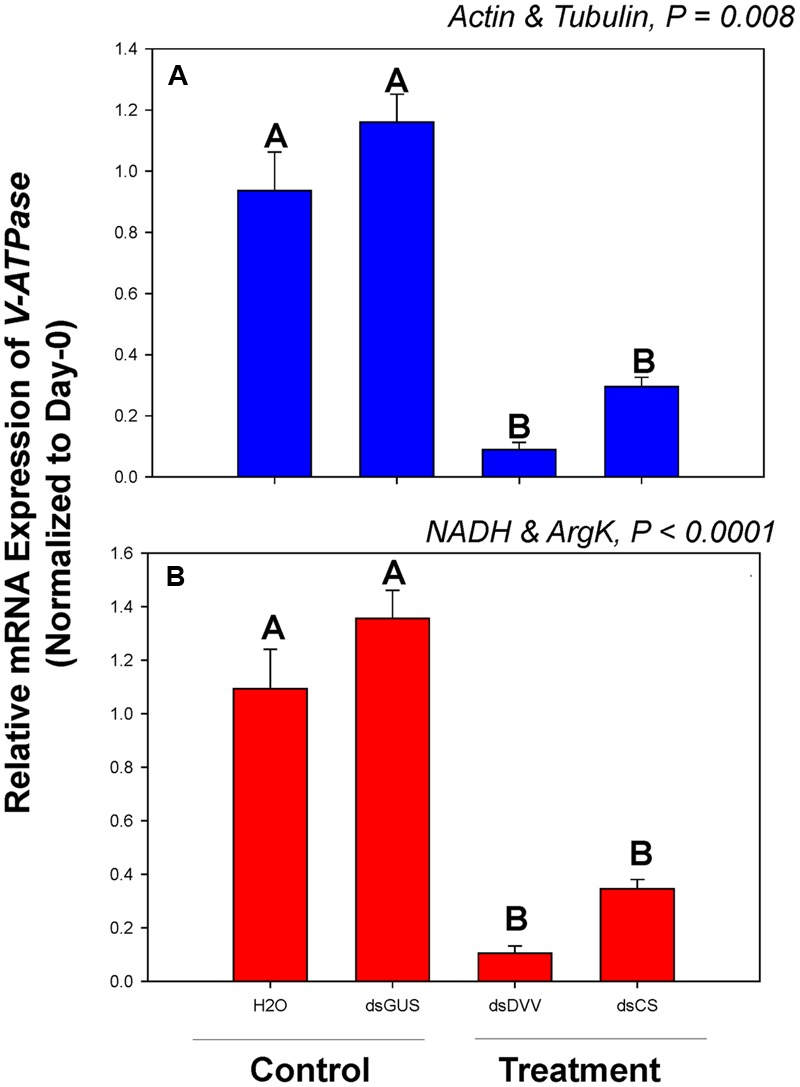
**Relative gene expression of the *V-ATPase* in *C. septempunctata* under dietary RNAi treatments.** The relative gene expression levels of *V-ATPase* were normalized to the most suited non-rRNA (**A**, *Actin* and *Tubulin*) and the least suited (**B**, *NADH* and *ArgK*) reference genes, respectively. For dietary RNAi, ladybeetle larvae were exposed to an artificial diet containing 15% sugar solution and 4.0 μg/μl dsRNAs for 2 days (see Materials and Methods for details). The transcript levels of *V-ATPase* in newly emerged (0 day) untreated larvae were set to 1, and the relative mRNA expression levels in dsRNA-fed larvae were determined with respect to the controls. Values are means ± SE. Different letters indicate significant differences between the treatments and controls (*P* < 0.05).

Among dsRNA treatments, *V-ATPase* expression differed when normalized to the two most- and least-stable non-rRNA reference genes (**Figure 4**). Expression of *V-ATPase* was most suppressed on day 3 in the dsDVV and dsCS treatments relative to the dsGUS and H_2_O controls (**Figure 4**).

**FIGURE 5:**
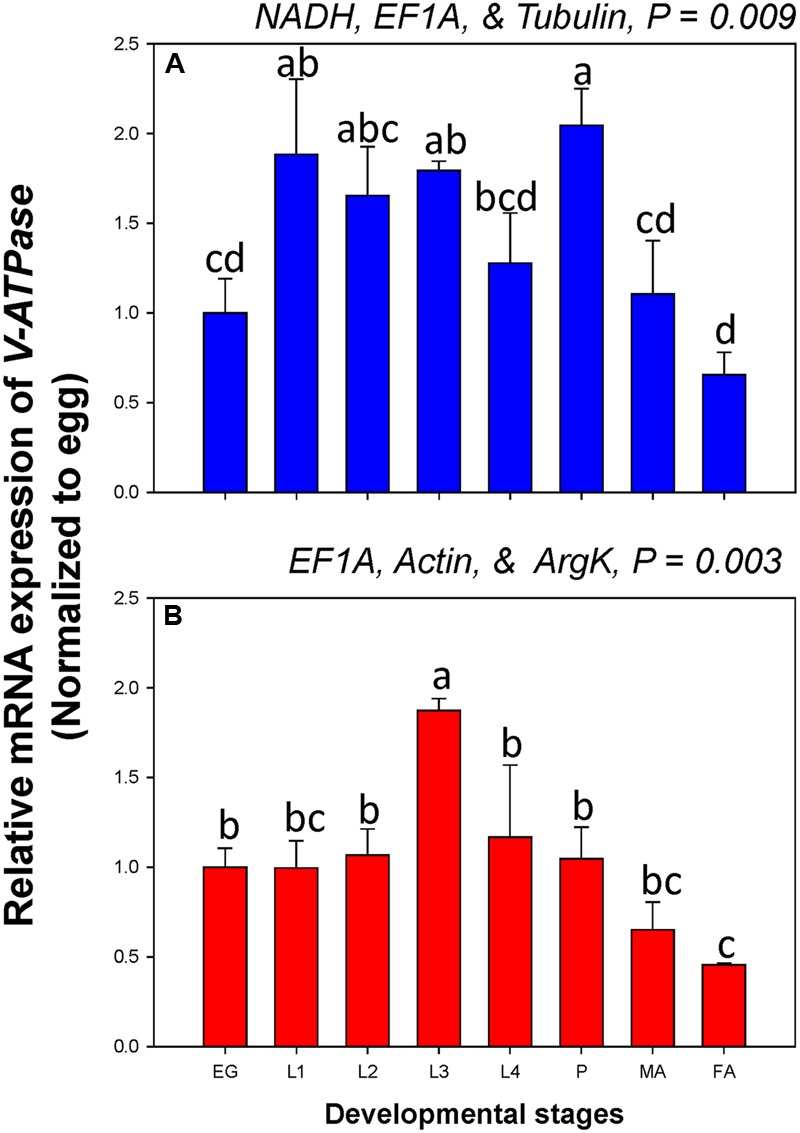
**Relative gene expression of the *V-ATPase* in different developmental stages of *C. septempunctata*.** The relative gene expression levels of *V-ATPase* in eggs (EG), first instar larvae (L1), second instar larvae (L2), third instar larvae (L3), fourth instar larvae (L4), pupae (P), male adults (MA), and female adults (FA) were normalized to the most suited non-rRNA (**A**, *NADH, EF1A*, and *Tubulin*) and the least suited (**B**, *EF1A, Actin*, and *ArgK*) reference genes, respectively. The relative expression level (fold) was calculated based on the value of the egg stage expression detected, which was assigned an arbitrary value of 1. Values are means ± SE. Different letters indicate significant expression differences among different developmental stages of *C. septempunctata* (*P* < 0.05).

Among developmental stages, *V-ATPase* expression differed when normalized to the three most- and least-stable non-rRNA reference genes. The expression level was different for each developmental stage under the two normalization conditions (**Figure 5**).

**FIGURE 6:**
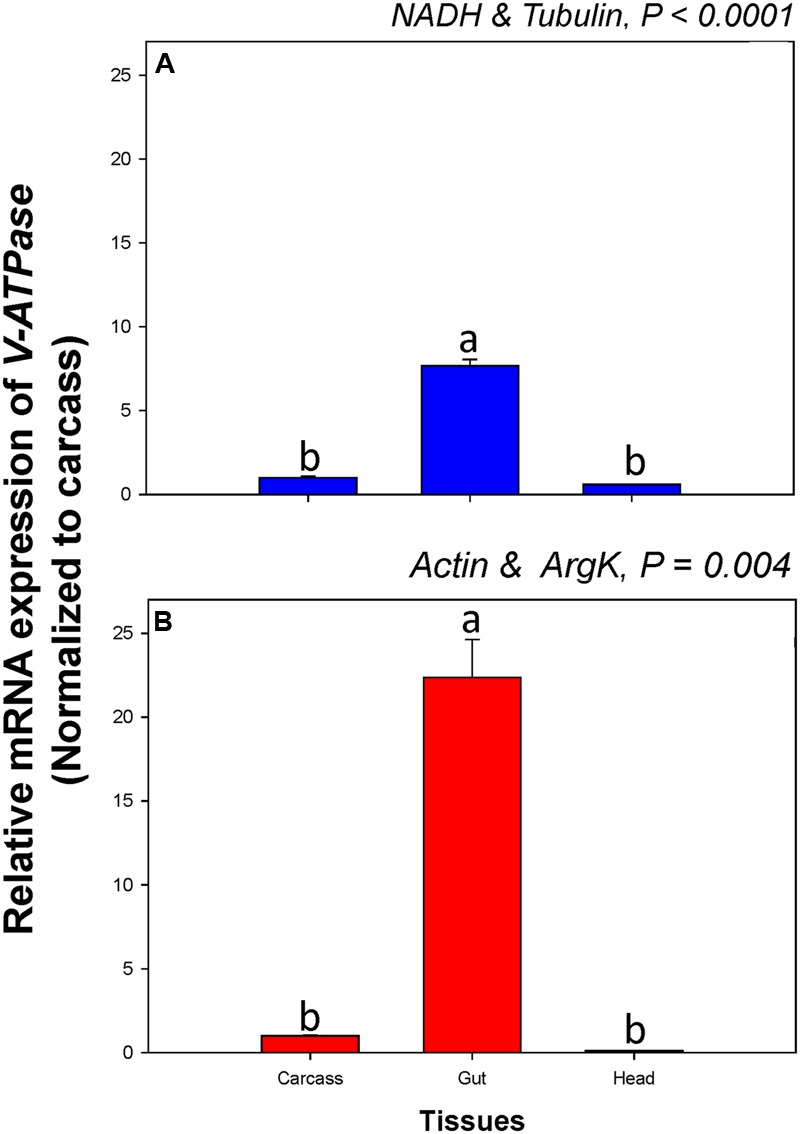
**Relative gene expression of the *V-ATPase* in different tissues of *C. septempunctata*.** The relative gene expression levels of *V-ATPase* were normalized to the most suited non-rRNA (**A**, *NADH* and *Tubulin*) and the least suited (**B**, *Actin*, and *ArgK*) reference genes, respectively. The relative expression level (fold) was calculated based on the value of the carcass expression detected, which was assigned an arbitrary value of 1. Values are means ± SE. Different letters indicate significant expression differences among different tissues of *C. septempunctata* (*P* < 0.05).

Among tissue types, *V-ATPase* expression was similar when normalized to the two most- and least-stable non-rRNA reference genes. When normalized to the most- and least stable reference genes, however, *V-ATPase* expression in the gut was about 7.7 and 22.4-fold higher, respectively, than in the carcass (**Figure 6**).

## Discussion

Although a wide range of techniques (e.g., cDNA microarray, subtractive hybridization, Western blot, Northern blot, RNA sequencing) can be used to study gene expression, the advantages of RT-qPCR in terms of its sensitivity and specificity, especially in non-model organisms, makes this an especially valuable tool. This technique, however, requires reference gene normalization to achieve reliable and comparable results (Kalushkov and Hodek, 2004; Li et al., 2013; Sinha and Smith, 2014; Zhu et al., 2014; Pan et al., 2015a; Wang et al., 2015). Our results confirm that the most stable reference genes can vary in different experimental conditions. While *V-ATPase* was the least stable *C. septempunctata* candidate gene in different tissues and dsRNA conditions, for example, it was very stable across different developmental stages. This is consistent with recent work suggesting that while *V-ATPase* was an unstable candidate gene for *Hippodamia convergens* (Coleoptera: Coccinellidae) in different tissue and dsRNA conditions, it was stable across developmental stages (Pan et al., 2015b).

The best-practice “Minimum Information for Publication of Quantitative Real-Time PCR Experiments” suggests using multiple reference genes in order to avoid biased normalization (Bustin et al., 2009). While two reference genes were adequate to analyze gene expression in different tissue and dietary RNAi conditions, we found that three reference genes were necessary across different developmental stages. The former result is consistent with previous work showing that two reference genes are sufficient for dependable gene normalization in different tissue and dietary RNAi conditions (Yang et al., 2015). The latter result, that more reference genes were required for developmental-stage analysis, likely reflects the fact that dramatic changes in gene expression occur during metamorphosis from one developmental stage to another (egg/larva, larva/pupa, pupa/adult). The *C*_t_-value of *Actin* is ∼24 at the egg stage, for example, and 16–19 at other developmental stages.

Levels of *V-ATPase* expression varied substantially following normalization to the most- and least-stable reference genes (**Figures 4–6**). This finding is consistent with previous work documenting condition-dependent variation in reference gene expression, and underlines the need for reference genes to be validated under particular experimental condition prior to their use (Bustin et al., 2013; Hellemans and Vandesompele, 2014; Yang et al., 2015).

Since rRNA makes up a large proportion of the total RNA pool (more than 80%), it is reflect by the three ribosomal genes (*18S, 28S*, and *16S*) showed mean *C*_q_-values less than 15 cycles, whereas mRNA makes up only 3–5%, thus, the use of rRNA for normalization of RT-qPCR may be problematic. Given this, it would have been preferable to include several mRNA species of the ribosomal machinery, e.g., *40S ribosomal protein S24, 40S ribosomal protein S18, 60S ribosomal protein L4* as opposed to only *18S, 28S*, and *16S*. Because many other studies still choose rRNA as the reference genes, however, we present the results of the three rRNA genes to illustrate how rRNAs are expressed under different experimental conditions in *C. septempunctata*. In addition, the least-expressed reference gene, like *ArgK* in *C. septempunctata* and *C. maculata* (Yang et al., 2015), is also not fit for the normalization.

This study, combined with our previous results, show that the three predatory ladybeetles, which share the same receiving environment (the maize field) and phylogenetically closely related to *D. v. virgifera*, are susceptive to ingested dsRNAs (Yang et al., 2015; Pan et al., 2015b). Additionally, dietary RNAi of dsDVV had no impacts on the other three NTOs including *Apis mellifera, Sinella curviseta*, and *Danaus plexippus* at both transcriptional and phenotypic levels (Pan et al., 2015a, 2016; Vélez et al., 2016). Our results are thus consistent with previous studies suggesting that the spectrum of dsRNA activity is expected to be narrow and species taxonomically related to the target organism are more likely to be susceptible (Baum et al., 2007; Bachman et al., 2013).

RNA interference has a wide range of applications in agriculture, especially for crop protection. Because many NTOs provide diverse ecosystem services (e.g., biological control, pollination, and decomposition), however, the effect of RNAi transgenic plants on species and the services they provide should be evaluated prior to commercialization. Our study thus provides a starting point for future work assessing the risks associated with RNAi transgenic plants. In addition, among different developmental stages and tissue types, *V-ATPase* expression differed when normalized to the two most- and least-stable non-rRNA reference genes, respectively. Expression of *V-ATPase* in the gut, for instance, ranged from 7.7 and 22.4-fold higher than in the carcass when normalized to the most- and least-stable sets of reference genes, respectively (**Figure 6**). Better accuracy in gene expression analysis not only can facilitate our investigation of gene function and evaluation of its efficacy for pest control, but also can improve the assessment of risks associated with RNAi transgenic plants.

Our results describe the selection and evaluation of stable *C. septempunctata* reference genes for use as internal controls in gene expression analysis. Eight endogenous reference genes were selected in 45 different samples for one abiotic (dietary RNAi) and two biotic (developmental stage and tissue type) conditions using five commonly used analytical methods. Different non-rRNA reference genes are recommended for each experimental condition: *NADH, EF1A*, and *Tubulin* across different development stages, *NADH* and *Tubulin* in different tissues, and *Tubulin* and *Actin* for the dietary RNAi experiment. Although the selection of reference genes is trait, species, condition and treatment specific, this study represents the first step toward establishing standardized RT-qPCR analysis in *C. septempunctata*. In addition, the methodology described here represents a critical step toward the development of an *in vivo* dietary RNAi toxicity assay for assessing the risks associated with RNAi transgenic plants.

## Author Contributions

XZ and HP conceived and designed research. HP, CY, and HZ conducted experiments. YL, LD, and XZ contributed reagents and analytical tools. HP and CY analyzed data. HP, EP, CY, and XZ wrote the manuscript.

## Conflict of Interest Statement

The authors declare that the research was conducted in the absence of any commercial or financial relationships that could be construed as a potential conflict of interest.

## Footnotes

1 http://blocks.fhcrc.org/codehop.html

2 https://www.idtdna.com/Primerquest/Home/Index

3 http://fulxie.0fees.us/?ckattempt=1

## Supplementary Material

FIGURE 1 **Alignment of *v-ATPase A* ORFs between *Coccinella septempunctata* and *Diabrotica virgifera virgifera*.** Identical nucleotides are highlighted in black boxes.

FIGURE 2 **The alignment of a highly conserved region within the ORFs of *v-ATPase A* from *C. septempunctata* and *Diabrotica virgifera virgifera*.** This 400 bp fragment was selected as the target template to synthesis insecticidal dsRNAs.

FIGURE 3 **The agrose gel profile of the eight candidate reference genes and one target gene in *C. septempunctata*.** M, EZ Load^TM^ 100 bp Molecular Ruler; Templates in the PCR reactions were as follows: (1) *28S*, (2) *18S*, (3) *16S*, (4) *EF1A*, (5) *Tubulin*, (6) *V-ATPase*, (7) *Actin*, (8) *NADH*, and (9) *ArgK*.

FIGURE 4 **Melting curves of the eight candidate reference genes and one target gene in *C. septempunctata***.

FIGURE 5 **Standard curves of the eight candidate reference genes and one target gene in *C. septempunctata***.
