# Corrigendum: Selection of Reference Genes for RT-qPCR Analysis in *Coccinella septempunctata* to Assess Un-intended Effects of RNAi Transgenic Plants

**DOI:** 10.3389/fpls.2016.01835

**Published:** 2016-11-29

**Authors:** Chunxiao Yang, Evan L. Preisser, Hongjun Zhang, Yong Liu, Liangying Dai, Huipeng Pan, Xuguo Zhou

**Affiliations:** ^1^College of Plant Protection, Hunan Agricultural UniversityChangsha, China; ^2^Institute of Plant Protection, Hunan Academy of Agricultural SciencesChangsha, China; ^3^Department of Entomology, University of KentuckyLexington, KY, USA; ^4^Department of Biological Sciences, University of Rhode IslandKingston, RI, USA; ^5^Institute for the Control of Agrochemicals, Ministry of AgricultureBeijing, China; ^6^Key Laboratory of Bio-Pesticide Innovation and Application of Guangdong Province, Department of Entomology, South China Agricultural UniversityGuangzhou, China

**Keywords:** *Coccinella septempunctata*, RT-qPCR, reference gene, RNAi transgenic plants, environmental risk assessment, plant incorporated protectant

Reason for Corrigendum:

In the original article, we neglected to mention the additional affiliation of Xuguo Zhou (^1^College of Plant Protection, Hunan Agricultural University, Hunan, China, and ^3^Department of Entomology, University of Kentucky, Lexington, KY, USA). The authors apologize for this oversight. This error does not change the scientific conclusions of the article in any way.

## Conflict of interest statement

The authors declare that the research was conducted in the absence of any commercial or financial relationships that could be construed as a potential conflict of interest.

