# Supplementary figures and images for: Selection of Reference Genes for RT-qPCR Analysis in Coccinella septempunctata to Assess Un-intended Effects of RNAi Transgenic Plants
Source: Front Plant Sci. 2016 Nov 8;7:1672. doi: 10.3389/fpls.2016.01672 (PMC5099537; doi:10.3389/fpls.2016.01672)

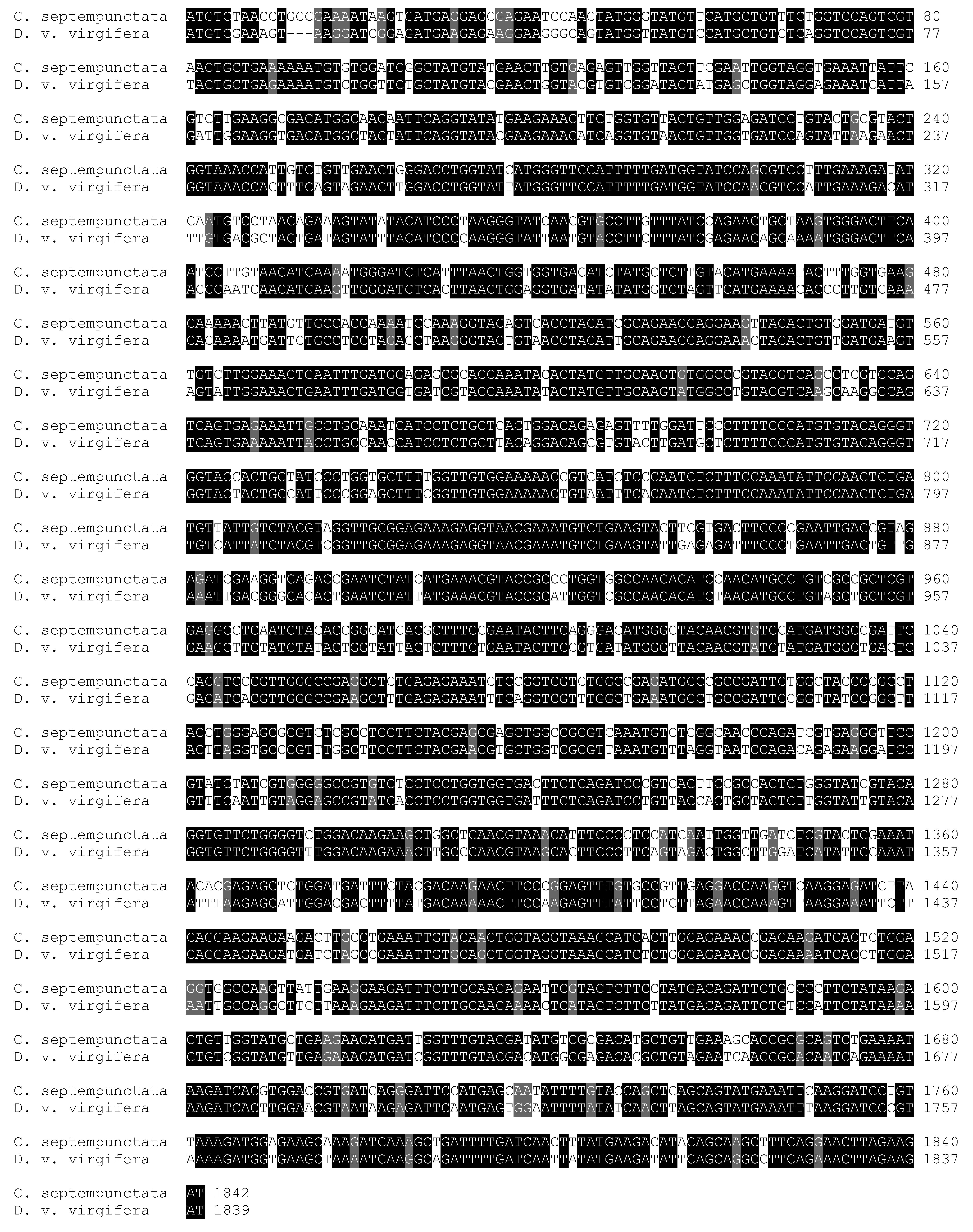

Supplement: FIGURE 1 — Alignment of v-ATPase A ORFs between Coccinella septempunctata and Diabrotica virgifera virgifera. Identical nucleotides are highlighted in black boxes. [file Image_1.TIFF]

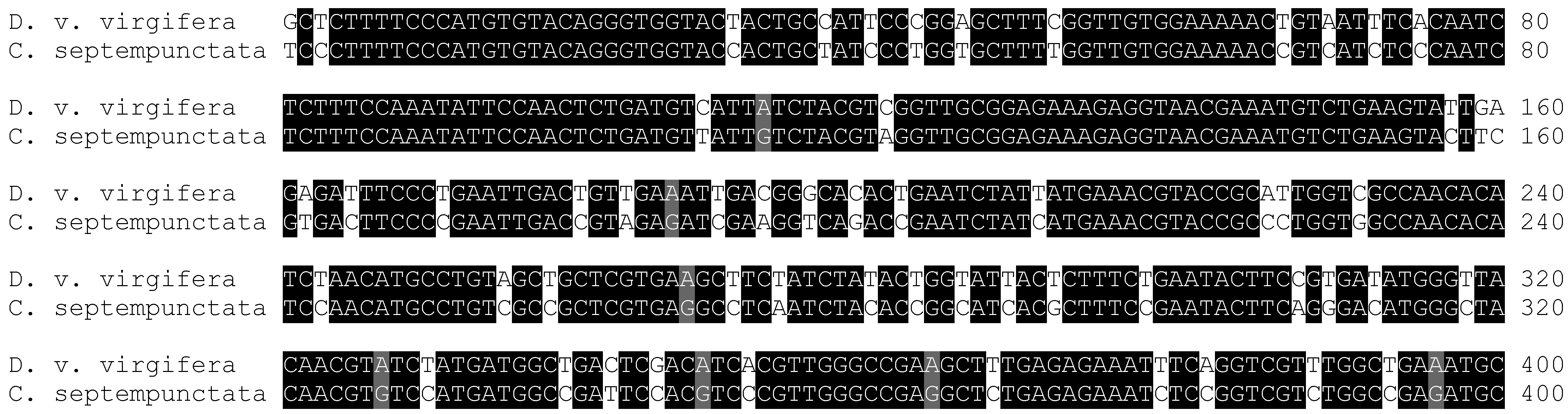

Supplement: FIGURE 2 — The alignment of a highly conserved region within the ORFs of v-ATPase A from C. septempunctata and Diabrotica virgifera virgifera. This 400 bp fragment was selected as the target template to synthesis insecticidal dsRNAs. [file Image_2.TIFF]

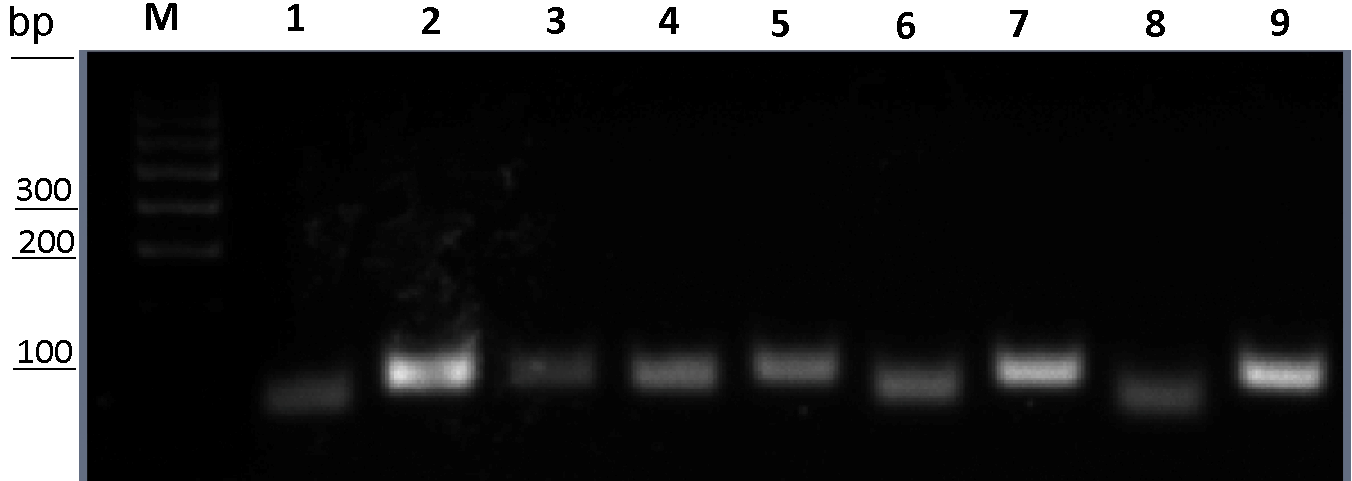

Supplement: FIGURE 3 — The agrose gel profile of the eight candidate reference genes and one target gene in C. septempunctata. M, EZ LoadTM 100 bp Molecular Ruler; Templates in the PCR reactions were as follows: (1) 28S, (2) 18S, (3) 16S, (4) EF1A, (5) Tubulin, (6) V-ATPase, (7) Actin, (8) NADH, and (9) ArgK. [file Image_3.TIFF]

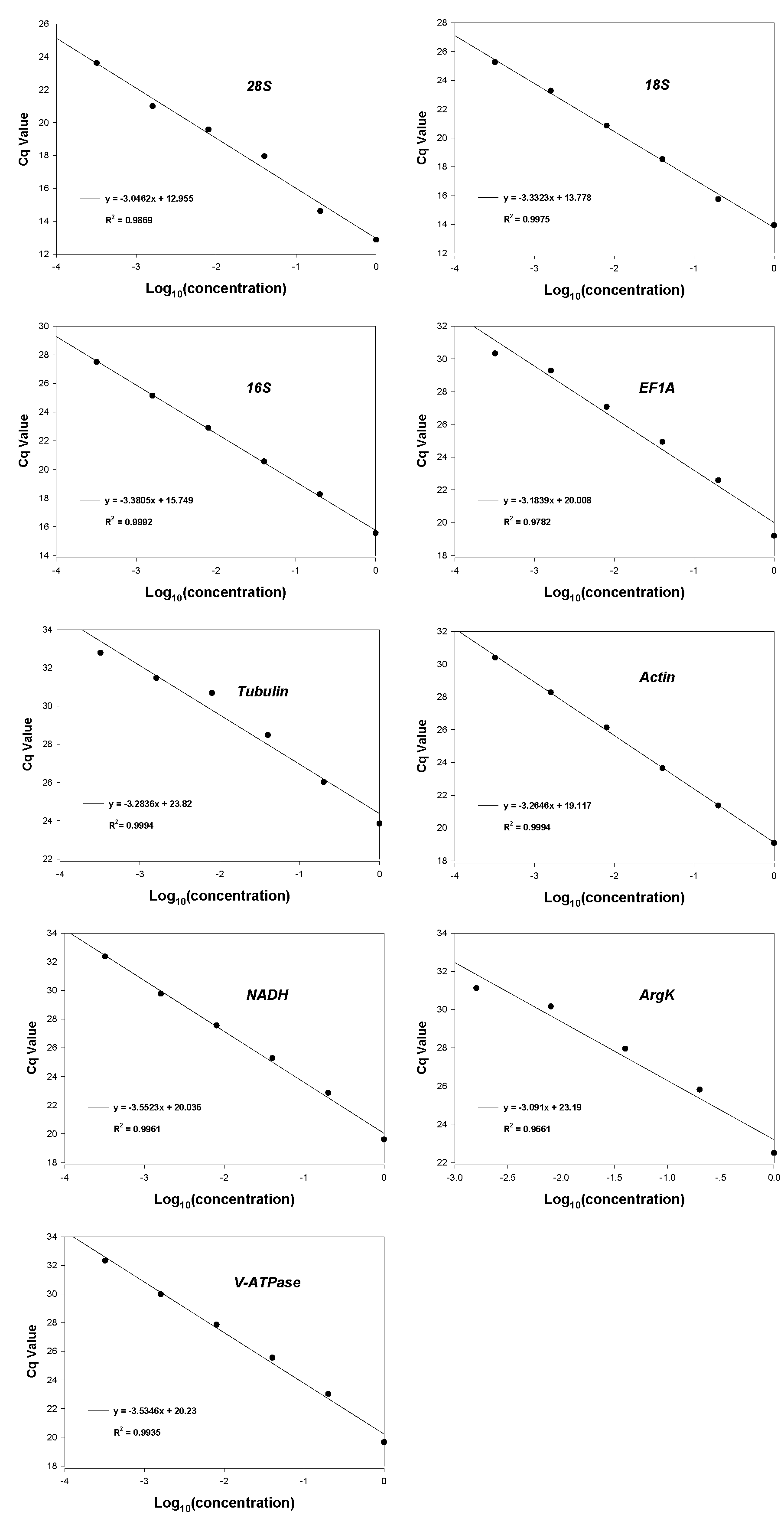

Supplement: FIGURE 4 — Melting curves of the eight candidate reference genes and one target gene in C. septempunctata. [file Image_4.TIFF]

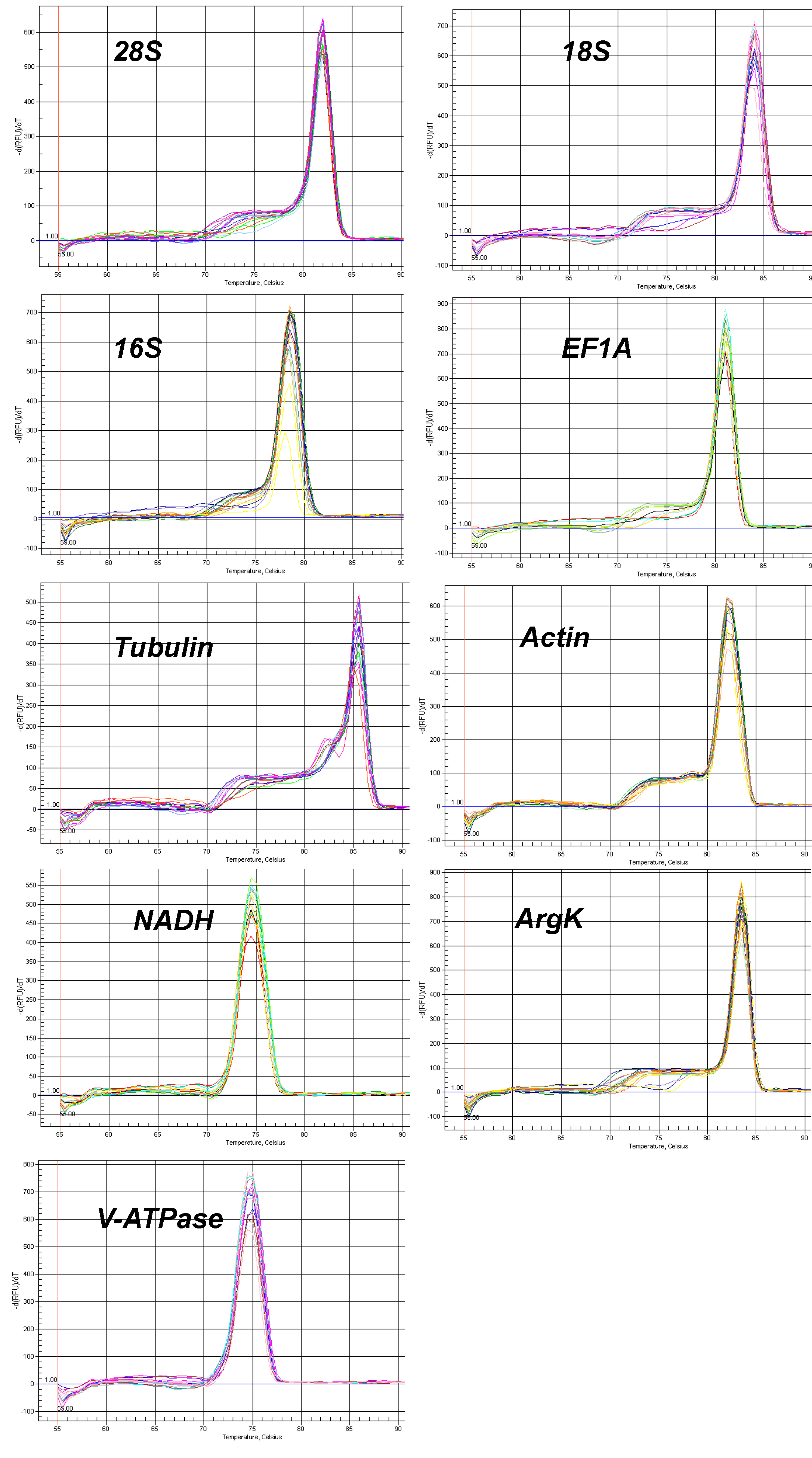

Supplement: FIGURE 5 — Standard curves of the eight candidate reference genes and one target gene in C. septempunctata. [file Image_5.TIFF]
