# Supplementary material for: Selection of Reference Genes for RT-qPCR Analysis in Coccinella septempunctata to Assess Un-intended Effects of RNAi Transgenic Plants
Source: Front Plant Sci. 2016 Nov 8;7:1672. doi: 10.3389/fpls.2016.01672 (PMC5099537; doi:10.3389/fpls.2016.01672)
Supplement: Supplementary file 6 [file Table_1.DOCX]

**Table S1. Degenerate primers used for amplification of four reference genes including *Actin*, *ArgK*, *EF1A*, *Tubulin*, and one target gene *V-ATPase***

| Gene | Primer sequences (5’-3’) |
| --- | --- |
| *Actin* | F: CGACATGGAAAAGATCTGGCAYCAYAC |
|  | R: TCGGTCAGCGATACCAGGRTACATNGT |
| *ArgK* | F:CCTGTTCGACCCTATCATCGARGAYTAYCA |
|  | R:GTCGTAGATACCACCTTCAGCTTCNGTRTGYTC |
| *EF1A* | F: TCGACATCGCTCTGTGGAARTTYGARAC |
|  | R: GTACCGATACCACCGATTTTGTANACRTCYTG |
| *Tubulin* | F: CTACAGGTTTCAAAGTGGGTATCAAYTAYCARCC |
|  | R: CCTTCGCCGACGTACCARTGNACRAA |
| *V-ATPase* | F: AGATGTCCGGATCNGCTATGTACGA |
|  | R: ACGAGCAGCCACAGGCATGTT |
