# Supplementary material for: Selection of Reference Genes for RT-qPCR Analysis in Coccinella septempunctata to Assess Un-intended Effects of RNAi Transgenic Plants
Source: Front Plant Sci. 2016 Nov 8;7:1672. doi: 10.3389/fpls.2016.01672 (PMC5099537; doi:10.3389/fpls.2016.01672)
Supplement: Supplementary file 7 [file Data_Sheet_1.DOCX]

**The sequence information of these four reference genes including *Actin*, *ArgK*, *EF1A*, *Tubulin*, and one target gene *V-ATPase***

>*Actin*

GGTCAGCGATACCAGGATACATCGTGGTACCACCAGAGAGTACGGTGTTGGCGTACAAGTCCTTACGGATATCAACGTCACACTTCATGATGGAGTTGTAGACGGTTTCGTGGATACCGCAGGATTCCATACCCAAGAAGGAAGGTTGGAAGAGGGCTTCTGGGCAACGGAATCTTTCGTTACCGATGGTGATCACTTGACCGTCAGGAAGTTCATAGGATTTTTCCAAGGAGGTGGAGGCAGCGGCAGTAGCCATTTCTTGTTCGAAGTCGAGGGCGACATAGCAAAGTTTTTCCTTGATGTCACGAACGATTTCCCTTTCAGCGGTGGTGGTGAATGAGTAACCCCTTTCGGTAAGGATTTTCATGAGGTAGTCGGTCAAGTCACGACCAGCCAAGTCAAGACGGAGGATGGCGTGGGGAAGGGCGTAACCTTCGTAGATTGGTACAGTGTGGGATACACCATCTCCAGAGTCCAAGACGATACCAGGGTACGACCGGAAGCATACAGGGAGAGTACAGCTTGGATGGCGACGTACATGGCTGGGGTGTTGAAGGTTTCGAACATGATTTGGGTCATCTTTTCTCTGTTGGCCTTGGGGTTCAATGGAGCTTCGGTCAAGAGGACTGGGTGTTCTTCTGGGGCAACACGAAGTTCATTGTAGAAGGTGTGATGCCAGATCTTTTCCATGTCG

>*ArgK*

GTCGTAGATACCACCTTCAGCTTCGGTATGTTCACCACATGTGCCACGTACCTGAAGATTGAACTTGCCGGCGATCTCGTCGAGCTTAGCCTTGTTAGAGGCCAATTTTGGCACCTTGATGTGAACAGAAGCCCTCACAGTGGTGCCCAAGTTGGTTGGACAGAAAGTGAGGAAACCCAGCCTGTCGTTGTGTGAAAATGGCAGACGTTTCTCGATTTCGTTAACAGCAGTAACAAGACGACGGTAAACTTGACCCAGGTCGCCTCCCATTTGCATCGAGATGATTCTGAGATGATCCTCTTCGTTGCACCAGACCAGGAAGGATTTGGCATCGTTATGGAATATTCCACGACCAGTTGGCCAGAATCTGCAGGCGTTTGCAGCCTGCAAGAATCTGTCTCCCTCCTTGAACAGGAAGTGATCGTCGATCAGCTTCTGCTGGGTTTCCTTGTCCATTCCAGTTAACGGATAGAAGGTACCTTTGAGTTCTCCATCAAGTCCAGACAGTGTGGATGAAACCTTCTGCTCCATCTCCTTGTATTGTTCTTCCGTTAAGCAAGGATTGAAGGGATAACCTTCCAAAGAACGACCGCATCTAACACGGGTAGATACGATGTATTCTCCAGCAGGATCCAAGTTGCCAAAAACATTCACATCCCCAAAATCTCTAAGGGGATGCTTGTCAGTCTTTTTGAAGCCACCATGATAATCTTCGATGATAGGGTCGAACAGG

>*EF1A*

GTACCGATACCACCGATTTTGTATACGTCCTGAAGTGGAAGACGAAGAGGTTTCTCGGTAGGACGAGATGGGGGAAGAATAGCGTCAAGAGCTTCAATCAAACATTTACCATCAGCCTTTCCTTCTTTACGTTCAATAGCCCATCCCTTGAACCATGGCATTTTGGTGGAAGGTTCCAACATGTTGTCTCCATGCCATCCAGAGATAGGCACAAAAGCTACAGCTGCAGGGTCGTAACCAATTTTTTTAATGTATGACGATACTTCCTTCCTGATTTCTTCGAAACGAGATTCGCTGTACGGTGGTTCAGTGGAGTCCATTTTGTTCACACCAACGATGAGCTGTTTTACTCCCAATGTGAAGGCGAGCAGAGCATGCTCACGAGTTTGTCCATTCTTCGAGATACCGGCTTCAAATTCACCAGTACCGGCAGCTACAATCAACACGGCACAATCTGCTTGAGATGTACCTGTGATCGTGTTTTTGATAAAATCTCTGTGTCCAGGGGCGTCAATAATGGTCACATAGTATTTGGAGGTTTCAAATTTCCACAGAGCGATGTCGA

>*Tubulin*

CTACAGGTTTCAAAGTGGGTATCAACTACCAGCCCCccACTGTGGTGCCGGGAGGAGACCTCGCCAAGGTACAGAGGGCGGTGTGCATGTTGTCAAACACCACCGCCATCGCTGAAGCCTGGGCCAGACTCGACCACAAGTTCGATCTCATGTACGCCAAGCGTGCCTTCGTCCACTGGTACGTCGGCGAAGG

>*V-ATPase*

AGATGTCCGGATCGGCTATGTACGAACTTGTGAGAGTTGGTTACTTCGAATTGGTAGGTG

AAATTATTCGTCTTGAAGGCGACATGGCAACAATTCAGGTATATGAAGAAACTTCTGGTG

TTACTGTTGGAGATCCTGTACTGCGTACTGGTAAACCATTGTCTGTTGAACTGGGACCTG

GTATCATGGGTTCCATTTTTGATGGTATCCAGCGTCCTTTGAAAGATATCAATGTCCTAA

CAGAAAGTATATACATCCCTAAGGGTATCAACGTGCCTTGTTTATCCAGAACTGCTAAGT

GGGACTTCAATCCTTGTAACATCAAAATGGGATCTCATTTAACTGGTGGTGACATCTATG

CTCTTGTACATGAAAATACTTTGGTGAAGCAAAAACTTATGTTGCCACCAAAATCCAAAG

GTACAGTCACCTACATCGCAGAACCAGGAAGTTACACTGTGGATGATGTTGTCTTGGAAA

CTGAATTTGATGGAGAGCGCACCAAATACACTATGTTGCAAGTGTGGCCCGTACGTCAGC

CTCGTCCAGTCAGTGAGAAATTGCCTGCAAATCATCCTCTGCTCACTGGACAGAGAGTTT

TGGATTCCCTTTTCCCATGTGTACAGGGTGGTACCACTGCTATCCCTGGTGCTTTTGGTT

GTGGAAAAACCGTCATCTCCCAATCTCTTTCCAAATATTCCAACTCTGATGTTATTGTCT

ACGTAGGTTGCGGAGAAAGAGGTAACGAAATGTCTGAAGTACTCCGTGACTTCCCCGAAT

TGACCGTAGAGATCGAAGGTCAAACCGAATCTATCATGAAACGTACCGCCCTGGTGGCCA

ACACATCCAACATGCCTGTGGCTG
